# Association of Fluorescent Protein Pairs and Its Significant Impact on Fluorescence and Energy Transfer

**DOI:** 10.1002/advs.202003167

**Published:** 2020-11-23

**Authors:** Jacob R. Pope, Rachel L. Johnson, W. David Jamieson, Harley L. Worthy, Senthilkumar Kailasam, Rochelle D. Ahmed, Ismail Taban, Husam Sabah Auhim, Daniel W. Watkins, Pierre J. Rizkallah, Oliver K. Castell, D. Dafydd Jones

**Affiliations:** ^1^ Molecular Biosciences School of Biosciences Cardiff University Cardiff CF10 3AX UK; ^2^ School of Pharmacy Cardiff University Cardiff CF10 3NB UK; ^3^ Present address: Henry Wellcome Building for Biocatalysis Biosciences University of Exeter Exeter EX4 4QD UK; ^4^ McGill University and Genome Quebec Innovation Centre Montreal Quebec H3A 0G1 Canada; ^5^ Department of Human Genetics McGill University Montreal Quebec Canada; ^6^ Department of Biology College of Science University of Baghdad Baghdad Iraq; ^7^ Present address: School of Biochemistry University of Bristol Bristol BS8 1QU UK; ^8^ School of Medicine Cardiff University Cardiff CF14 4XN UK

**Keywords:** fluorescence, fluorescent proteins, Förster resonance energy transfer (FRET), oligomerization, protein design

## Abstract

Fluorescent proteins (FPs) are commonly used in pairs to monitor dynamic biomolecular events through changes in proximity via distance dependent processes such as Förster resonance energy transfer (FRET). The impact of FP association is assessed by predicting dimerization sites in silico and stabilizing the dimers by bio‐orthogonal covalent linkages. In each tested case dimerization changes inherent fluorescence, including FRET. GFP homodimers demonstrate synergistic behavior with the dimer being brighter than the sum of the monomers. The homodimer structure reveals the chromophores are close with favorable transition dipole alignments and a highly solvated interface. Heterodimerization (GFP with Venus) results in a complex with ≈87% FRET efficiency, significantly below the 99.7% efficiency predicted. A similar efficiency is observed when the wild‐type FPs are fused to a naturally occurring protein–protein interface system. GFP complexation with mCherry results in loss of mCherry fluorescence. Thus, simple assumptions used when monitoring interactions between proteins via FP FRET may not always hold true, especially under conditions whereby the protein–protein interactions promote FP interaction.

## Graphical abstract

Fluorescent proteins (FP) are an important tool in the life sciences, including monitoring dynamic processes such as protein–protein interactions. Here, it is shown that when FPs associate their core functional parameters can change. Thus, simple assumptions used when monitoring interactions between proteins via FRET may not always hold true, especially under conditions whereby the protein–protein interactions promote FP interaction.

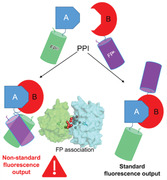

## 1 Introduction

Fluorescent proteins (FPs) have revolutionized biology through their use as genetically encoded imaging tags and biosensors.^[^ ^1^, ^2^, ^3^ ^]^ The subsequent engineering of a small subset of natural FPs,^[^ ^1^ ^]^ especially green fluorescent protein (GFP) from *Aequorea victoria* ^[^ ^4^ ^]^ and DsRed from coral^[^ ^3^ ^]^ have expanded their use by changing their spectral (e.g., *λ*_max_, *λ*_EM_, quantum yield, brightness) and structural (e.g., quaternary structure, stability, folding kinetics, chromophore maturation kinetics) properties. Many fluorescent proteins, especially those that emit in the red region, naturally exist as oligomers^[^ ^5^ ^]^ or have a tendency to oligomerize.^[^ ^6^ ^]^ There has been a great deal of protein engineering effort to generate functional monomeric forms but many commonly used FPs have a capacity to dimerize.^[^ ^6^, ^7^ ^]^ Dimerization can be compounded by local high concentrations brought about by interactions between the fusion partner proteins that are the prime focus of such studies. Yet there is little information concerning how oligomerization influences inherent function; oligomerization potential and functional impact is especially important for studies involving FPs pairs, such as Förster resonance energy transfer (FRET).

FRET is one of the most important applications of FP pairs as it can be used to monitor dynamic biological events such as protein–protein interactions.^[^ ^8^, ^9^ ^]^ FRET is largely a passive process that relies on two FPs with mutually compatible spectral properties (acceptor FP absorbance overlapping with donor FP emission wavelength) being in close proximity but not physically interacting; changes in distance between the two FPs changes efficiency of FRET between the donor and acceptor. Despite FRET being a mainstay of biomolecular interaction analysis, there are a several assumptions required such as freely rotating FPs that do not interact or align in any significant manner. Here, we show that both these factors may not always hold true.

As well as absolute distance between the FPs, the angular vector between the chromophore dipoles is critical; this is *κ* ^2^ value in Equation (1).

(1) $$\begin{array}{c} R_{0}=0.211\sqrt[{6}]{k^{2}n^{-4}Q_{D}J\left(\lambda \right)} \end{array}$$

where *R*_0_ is the Förster radius, *κ* ^2^ is the dipole orientation factor, *n* is the solvent refractive index, *Q*_D_ is the quantum yield of the donor, and *J*(*λ*) is the overlap integral between the donor emission and acceptor molar absorbance. *R*_0_ is used as a constant to relate energy transfer efficiency to distance between individual components via Equation (2).

(2) $$\begin{array}{c} r=R_{0}\sqrt[{6}]{\left(1-E\right)/E} \end{array}$$

where *r* is the distance between two FRET chromophores and *E* is the observed FRET efficiency. Critically *κ* ^2^ is arbitrarily set to 0.667 to reflect two randomly orientated chromophores as the transition dipole moment (TDM) arrangement is largely unknown, which in turn impacts on the calculated *R*_0_. In reality the two chromophores are unlikely to be truly freely rotating with respect to each other when fused to a protein of interest.^[^ ^9^ ^]^ Therefore, it is difficult to accurately equate FRET efficiency to distance. Furthermore, FP pairs may (and do^[^ ^6^ ^]^) physically interact which can result in changes in inherent function.^[^ ^10^, ^11^ ^]^ Thus, when investigating FRET between FPs there may not just be simple spatial proximity at work but molecular interactions leading to more defined distance and dipole alignment, which may in turn influence inherent fluorescence. It has previously been thought that by using FPs from different organism classes with low sequence identities (e.g., GFP with RFPs) should prevent dimerization.

**Figure 1:**
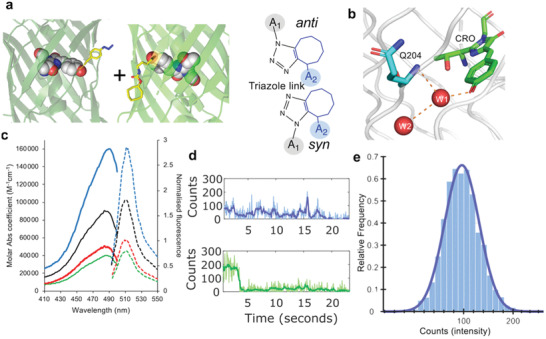
Click‐based protein dimerization via residue 204. a) Covalent crosslink via genetically encoded *p*‐azido‐L‐phenylalanine (azF) in one monomer and strained‐cyclooctyne pyrrolysine (SCO‐K) placed in the second monomer. Shown are the two different final regio‐isomers. b) Relative positioning of residue Q204 with respect to chromophore (CRO) and local water molecules (red spheres W1 and W2). c) Steady state bulk absorbance (full line) and fluorescence emission (dashed line) of sfGFP^204x2^ (blue), sfGFP^204azF^ (green), sfGFP^204SCO^ (red) and the addition of the two monomer spectra (black). The data has been reported previously^[^ ^11^ ^]^ and shown here for context. See Table S1, Supporting Information, for further spectral properties. d) Representative single molecule traces for sfGFP^204x2^ (blue) and sfGFP^WT^ (green) measured by TIRF microscopy. Further example of single molecule traces can be found in Figure S1, Supporting Information, for sfGFP^204x2^. Equivalent single molecule analysis of sfGFP^WT^ is described previously by Worthy et al.^[^ ^11^ ^]^ e) A single molecule fluorescence intensity histogram for sfGFP^204x2^ consisting of 179 trajectories (2602 spots). The histogram data fit to a single log normal distribution centered around 100 counts.

We^[^ ^11^ ^]^ and others^[^ ^12^, ^13^, ^14^, ^15^ ^]^ have previously shown that FP association can be promoted through either connecting FPs with linker sequences/protein domains, or by forming oligomers from individual monomers. In relation to the current work, we have shown that potential naturally occurring FP dimer interfaces can be predicted in silico and then stabilized via genetically encoded strain‐promoted azide‐alkyne cycloaddition (SPAAC);^[^ ^11^ ^]^ dimerization results in changes to the spectral properties. Here, we describe the construction and analysis of various SPAAC linked FP dimers (**Figure** **1**a). The structure of a super‐folder GFP (sfGFP) homodimer provides a rationale for enhanced fluorescence and the role of dynamics in this process. Using this new structural information, we determined *κ* ^2^ values and measured *J*(*λ*) to calculate more realistic *R*_0_ values for experimentally analyzed click linked sfGFP‐Venus dimers. We find that theoretical FRET efficiency does not match the observed FRET efficiency suggesting that proximity and dipole arrangement may not be the only factors that influence energy transfer. Furthermore, we linked sfGFP and mCherry together and found little FRET between the two proteins, with mCherry fluorescence being largely lost on dimerization.

## 2 Results and Discussion

### 2.1 The Effect of sfGFP Association on Function

We have previously reported the construction of FP dimers stabilized by click chemistry through the covalent coupling of genetically encoded ring‐strained cyclooctyne derivative of the pyrrolysine (SCO‐K) and *p‐*azido‐L‐phenylalanine (azF)^[^ ^11^ ^]^ (Figure 1a). It should be noted that we do not attempt to change FP dimer interface as a whole nor link them in a tandem arrangement using a spacer sequence as has been done in other approaches^[^ ^12^, ^13^, ^14^, ^15^, ^16^ ^]^ but model potential naturally occurring interface sites, which are in turn stabilized through an SPAAC link. Regions that do not naturally associate do not promote covalent crosslinking via SPAAC.^[^ ^11^ ^]^ Thus, our approach stabilizes naturally feasible protein interactions.

Residue Q204 is a surface exposed residue that lies close to the sfGFP chromophore (CRO; Figure 1b), with the backbone amine group making an indirect H‐bond with CRO via a conserved structured water molecule, W1. In silico, molecular docking^[^ ^11^ ^]^ revealed that Q204 consistently resided at possible dimer interfaces and is close to a region known to be involved in FP dimerization.^[^ ^7^ ^]^ The SCO‐K (sfGFP^204SCO^) and azF (sfGFP^204azF^) containing monomers were subsequently proved to dimerize, generating the dimer termed sfGFP^204x2^.^[^ ^11^ ^]^ The sfGFP 204‐linked dimer displayed enhanced fluorescence compared to the monomers. Dimeric sfGFP^204x2^ displayed positive functional synergy in which the brightness of the complex was more than the sum of the individual monomers (Figure 1c; Table S1, Supporting Information).^[^ ^11^ ^]^ Indeed, sfGFP^204x2^ has a greater brightness on a per CRO basis (56 800 M^−1^ cm^−1^) compared to the original sfGFP (36 750 M^−1^ cm^−1^)^[^ ^17^, ^18^ ^]^ and EGFP (34 650 M^−1^ cm^−1^).^[^ ^19^ ^]^ This change in fluorescence behavior is further confirmed at the single molecule level where the dimeric fluorophore is much more resistant to photobleaching, displaying longer on times compared to sfGFP^WT^ (average 0.87s GFP^204x2^ compared to 0.65s for GFP^WT^). (Figure 1d with additional traces in Figure S1, Supporting Information; see Worthy et al. for sfGFP^WT^ single molecule analysis^[^ ^11^ ^]^).

Single molecule fluorescent traces of the dimer are more complex and dynamic compared to sfGFP^WT^ with a range of dynamically fluctuating fluorescent intensities observed, not well described by discrete states (Figure 1d; Figure S1, Supporting Information), which could indicate cooperative interaction between the individual monomer units. Such communication between conjoined CROs is further exemplified in the counter‐intuitive ensemble fluorescence behavior of equivalently linked fluorescent protein heterodimers (vide infra). In the case of sfGFP^204x2^, the single molecule fluorescence time course traces are not consistent with expectations of two independent, co‐localized fluorophores, with an absence of two‐step photobleaching observed. Intensity state histograms compiled from single molecule traces reveal a single dominant intensity peak observed at a value similar to monomeric sfGFP^WT^ (Figure 1e). This is in contrast to the expected bimodal distribution of two independent fluorophores. If the two molecules in the dimer are acting largely independently of each other, a bimodal distribution would be expected arising from the combination of additive intensities of the two fluorophore states at any one time (i.e., ON/ON [2× intensity], OFF/ON and ON/OFF [1× intensity]). Consequently, the observed data is consistent with only 1 CRO in the dimer being fluorescent at any given time, and suggestive of a possible dependent activity relationship. The observations from single molecule and ensemble data suggest that the change in absorbance coefficient, increased resistance to photobleaching, and change in photo‐dynamics observed as intensity fluctuations at the single molecule level, collectively give rise to an overall increase in ensemble fluorescence.

### 2.2 Structural Basis for Association‐Based Effects

**Figure 2:**
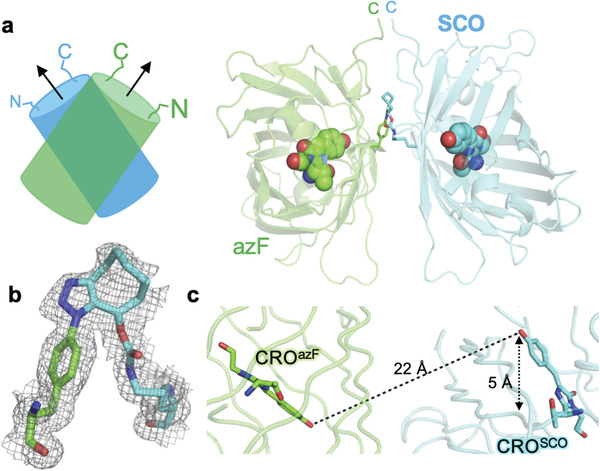
Structure of sfGFP^204x2^ (PDB 5NI3). a) Arrangement of the azF (green) and SCO (blue) containing monomers. b) The *syn* conformation of the triazole linkage with the electron density map (2Fo‐Fc, 1.0 sigma) shown. c) Distances and offset of the two CROs (shown as sticks).

The structure of sfGFP^204x2^ (structural statistics in Table S2, Supporting Information) reveals that each monomer unit is similar to the original starting sfGFP (Figure S2a,b, Supporting Information), with the chromophore retaining planarity in each monomer unit of the dimer. The sfGFP^204x2^ dimer forms a quasi‐symmetrical off‐set “side‐by‐side” monomer arrangement (**Figure** **2**a), which is promoted by formation of a syn 1,5 triazole link that generates a reverse turn structure (Figure 2b). The two CROs point toward each other in an antiparallel arrangement 22 Å apart with a 5Å offset (Figure 2c). It is closest to the third ranked in silico model predicted previously^[^ ^11^ ^]^ (Figure S2c, Supporting Information). Each monomer is offset by 70° with the C‐termini close in space (Figure 2b). As the N‐ and C‐termini are close to each other at the same end of the *β*‐barrel, the proximity and orientation of the two termini in the dimer may well promote such an interaction in a fusion protein construct.

**Figure 3:**
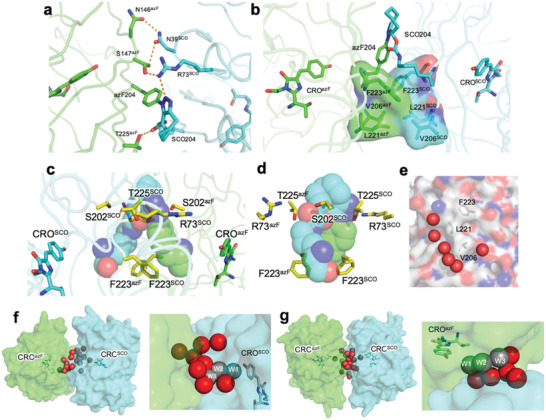
Subunit interface in sfGFP^204x2^ comprised of sfGFP^204azF^ (green) and sfGFP^204SCO^ (cyan). a) H‐bond network at interface. b) Hydrophobic interactions. c,d) Interactions around the triazole link shown in two different orientations. e) Water molecules (red spheres) associated with the interface region. Water‐rich cavities at dimer interface shown from two different angles. Waters (red spheres) associated with the f) sfGFP^204SCO^ CRO and g) sfGFP^204azF^ CRO. The grey spheres are equivalent to W1, W2, and W3 shown in Figure 1 and Figure S4, Supporting Information. Waters molecules W2 and W3 are observed in the sfGFP^WT^ structure but are largely surface exposed (Figure S4, Supporting Information).

The two monomer units associate to form an extensive (≈900 Å^2^) and intimate interface. The main elements that comprise a natural protein–protein interface, namely hydrophobic interactions and H‐bonding are observed (**Figure** **3**). The H‐bond network at the interface is not symmetrical but the hydrophobic interactions show a significant degree of symmetry (Figure 3a). The interlocking hydrophobic interface is comprised of Phe223, Val206, Leu221 from both chains (Figure 3b). These residues are surface exposed in sfGFP and form a naturally occurring hydrophobic patch^[^ ^7^ ^]^ that can facilitate and stabilize the dimer on click crosslinking (Figure 3e), or for that matter potentially other FPs. Indeed, mutation of Val206 to a charged residue is known to reduce dimerization tendency of *A. victoria* derived GFPs.^[^ ^7^ ^]^

The new triazole crosslink is integrated within the structure being semi‐buried at the dimer interface and lying above the plane of the main hydrophobic interface patch (Figure 3c,d). The azF component is buried while one face of the SCO moiety is partially accessible to the solvent. Phe223 from both monomers forms the base of the triazole reverse turn (Figure 3c,d) while Arg73, Ser202, and Thr225 residues make putative polar interactions with oxygen and nitrogen atoms in the SCO‐azide link. A more extended network linking the two chromophores is proposed in Figure S3, Supporting Information.

At the interface are two cavities filled with ordered water molecules (Figure 3f,g). The water molecules are arranged around an area where the chromophore protrudes toward the surface. A partially buried water molecule (W1; Figure 1c; Figure S4, Supporting Information) is commonly observed associated with the chromophore via a H‐bond with the phenol hydroxyl group and the backbone of residue 204; this water is associated with 1 to 2 additional surface water molecules (grey spheres, Figure 3f,g) as observed for monomeric sfGFP^WT^ (Figure S4, Supporting Information). In the sfGFP^204x2^ dimer, these waters lie within the cavity together with several additional tightly packed water molecules.

Generation of the dimer is likely to impact on both protein and solvent dynamics, which can in turn be expected to affect functional properties such as molar absorbance and brightness.^[^ ^20^ ^]^ Analysis of B‐factors suggest that regions containing key functional residues such as S205 and E222 become less dynamic on dimerization (Figure S5, Supporting Information). E222 also adopts a different side‐chain configuration in the dimers compared sfGFP^WT^. H148, another residue critical to function, is known to be dynamic, existing in “open” and “closed” conformation.^[^ ^17^, ^21^ ^]^ Only in the closed conformation can H148 help contribute to chromophore deprotonation, which in turn promotes absorbance at ≈490 nm. H148 is also located close to the dimer interface and occupies the closed configuration (Figure S5, Supporting Information). As well as changes to protein dynamics, the burial of waters at the interface is also likely to be important for enhanced function. The roles of the additional waters associated with W1 in terms of their impact on the structure–function relationship is not fully known but it has been postulated that they contribute to charge transfer and modulation of the protonated state of the CRO.^[^ ^22^ ^]^ In solution, it is likely that the additional water molecules associated with W1 are in free exchange with the solvent when sfGFP is monomeric; exchange with bulk solvent is likely to be reduced in the dimeric sfGFP^204x2^ so persist in a defined arrangement for longer. Thus, dimerization is likely to lead to a more rigid structure around the chromophore with more persistent bond networks and less conformational flux, with these stabilized interactions potentially contributing to the positive synergistic effect on brightness.

### 2.3 Heterodimers and Functional Communication by Energy Transfer

**Figure 4:**
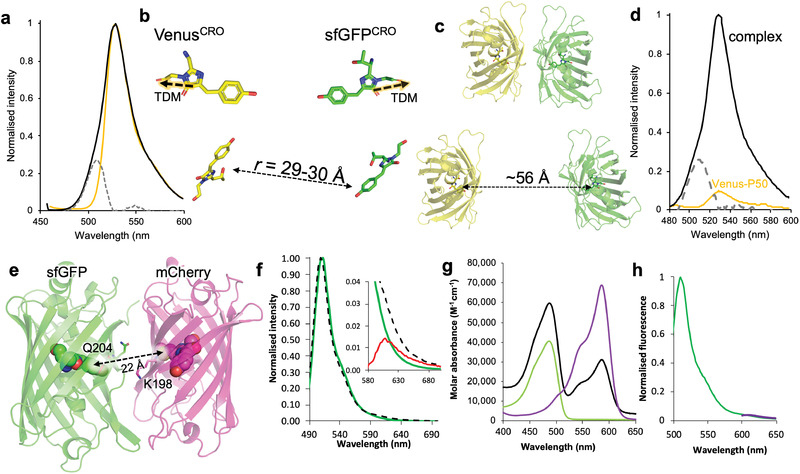
Heterodimer fluorescence characteristics. a) Fluorescence emission spectra of GFVen^204^ (black), Venus^204azF^ (gold), and the residual emission profile highlighting sfGFP contribution to GFVen^204^ spectrum (grey dashed). Excitation was at 450 nm. b) Relative positioning of the Venus (yellow) and sfGFP (green) chromophores (CROs) in the model GFVen^204^ structure. The dashed lines highlighted in orange represent the transition dipole moment (TDM). c) Relative distances between Venus (yellow) and sfGFP (green) based on the model of SPAAC linked dimer (top) and implied spacing calculated from observed FRET efficiency (bottom). Structures are to scale. d) FRET between sfGFP‐Bcl3 and P50‐Venus on excitation at 420 nm (black line), fluorescence due to P50‐Venus alone (gold line), and residual sfGFP‐Bcl3 fluorescence in the complex (grey dashed line). e) Modeled docking of mCherry (PDB:2H5Q) and sfGFP (PDB:2B3P) using ClusPro.^[^ ^23^ ^]^ Residues Q204 (sfGFP) and K198 (mCherry) that are replaced by SCO‐K and azF, respectively, are highlighted. f) Fluorescence emission (on excitation at 485 nm) of sfGFP^204SCO^ (green solid line) and GFCh^x2^ dimer (dashed black line). Emission intensities are normalized to sfGFP^204SCO^. Inset is the zoomed in region of the emission spectrum centered around 610 nm, the emission maximum of mCherry. The red corresponds to the subtraction of GFCh^x2^ from sfGFP^204SCO^. g) Molar absorbance of sfGFP^204SCO^ (green; *λ*_max_ 488 nm), mCherry^198azF^ (purple; *λ*_max_ 587 nm), and GFCh^x2^ (black line; *λ*_max_ 488 nm and *λ*_max_ 587 nm). h) Emission profile of GFCh^x2^ on excitation at 485 nm (green) and 585 nm (purple). Spectral properties are detailed in Table S1, Supporting Information.

The use of different FPs with compatible spectral properties to promote FRET is essential for biomolecular analysis. The sfGFP^204SCO^ variant can be linked to Venus (containing azF) via residue 204 to generate heterodimers.^[^ ^11^ ^]^ The resulting dimer, termed GFVen^204^, demonstrated FRET from the sfGFP component to Venus, as would be expected (**Figure** **4**a). There is currently very little known about the relative orientation of FRET‐based FP pairs with only one structure available in a biosensor configuration,^[^ ^12^ ^]^ which is in a single polypeptide format rather that a classical two‐protein system. Given the high degree of sequence and structure similarity between sfGFP and Venus, we used the GFP^204x2^ structure to build models of the GFVen^204^ dimer so as to calculate more specific *R*_0_ factors based on the relative orientations of the two chromophores (Figure S6, Supporting Information). Using our model of GFVen^204x2^, together with the dipole arrangements of known transitions for both GFP and Venus^[^ ^24^, ^25^ ^]^ (Figure 4b), *κ* ^2^ was calculated in the model to be 3.59. Using the *Q*_D_ and *J*(*λ*) values (Table S3, Supporting Information) together with a refractive index of 1.4 to account for a combined protein–water environment (Hellenkamp et al.^[^ ^9^ ^]^ and Dr. Tim Craggs personal communication) we calculated *R*_0_ with the different *κ* ^2^ values (Table S3, Supporting Information). The calculated *R*_0_ was ≈76 Å, which is up to 19 Å longer compared to when the arbitrary 0.667 *κ* ^2^ value is used. Our calculated *R*_0_ values are consistent with those calculated using *J*(*λ*) and donor QY values available through FPbase (https://www.fpbase.org)^[^ ^26^ ^]^ when adjusted for *κ* ^2^ (see Table S3, Supporting Information).

The question arises is how does our calculated *R*_0_ relate through to observed FRET efficiency. Based on the use of Equation (2) and the measured inter‐chromophore distance of 29–30 Å (Figure 4b), the estimated FRET efficiency for our GFVen^204^ construct should be close to 100% (99.6–99.7%). However, deconvolution of the GFVen^204^ emission on excitation at 450 nm (a wavelength that will excite predominately sfGFP) reveals a significant sfGFP component (Figure 4a). Using a commonly used approach based on Equation (3), apparent relative FRET efficiency can be calculated.

(3) $$\begin{array}{c} E_{\mathrm{rel}}=I_{A}/\left(I_{D}+I_{A}\right) \end{array}$$

where *E*_rel_ is relative FRET efficiency, *I*_A_ is integrated fluorescence of the acceptor, and *I*_D_ is the integrated fluorescence of the donor. The relative FRET efficiency was determined to be 87%. While donor (sfGFP^204SCO^) and acceptor (Venus^204azF^) quantum yield are likely to vary between the monomers and dimer (see Table S1, Supporting Information), even taking into consideration such variation in quantum yield, FRET efficiency is the range of 85–91%. Thus, there is a clear discrepancy between the observed and theoretical FRET efficiency, as has been observed before form structure‐based analysis where inter‐FP interactions were observed.^[^ ^12^ ^]^

The exact reason for the lower than expected FRET efficiency is not entirely clear. A simple and obvious explanation is that some free monomeric sfGFP^204SCO^ is present. Analysis of polyacrylamide gels and mass spectrum suggests little or no monomeric protein is present (see Worthy et al.^[^ ^11^ ^]^ and Figure S7, Supporting Information, for details). Are the considerable number of water molecules present at the domain interface observed for sfGFP^204x2^ (Figure 3f,g) playing a role in quenching? Water can quench fluorescence,^[^ ^4^, ^27^ ^]^ especially if collisional events are promoted through free dynamic exchange. However, the crystal structure suggests local water molecules are likely to be less dynamic in the dimer compared to monomeric forms. Furthermore, if water quenching was significant then the residual donor sfGFP signal would not be observed. Is the arrangement of the monomers in GFVen^204x2^ similar to the assumed sfGFP^204x2^? One possibility is that there is a mixed population of dimers; a major population in which FRET is highly efficient and a second minor conformation in which energy transfer from sfGFP to Venus is negligible. While modeling predicts GFVen^204^is likely to occupy a conformation similar to sfGFP^204x2^ we cannot rule out a minor state in which energy transfer is compromised. For example, a major shift in transition dipole arrangement could results in *κ* ^2^ being close to 0 or the chromophore conformation being altered (e.g., *cis*–*trans* isomerization). While we cannot rule out some rotation of one FP with respect to another, the triazole link should restrict such rotation and the CROs should retain a similar vector configuration in terms of the transition dipole moments. With an *R*_0_ of 76.62 Å, the two CROs will need to be at least 50 Å apart (shown schematically in Figure 4c) for FRET efficiency to be close to that observed. Even using the arbitrary *κ* ^2^ value of 0.667 produces an *R*_0_ of 56 Å, which will require the CROs to be ≈40 Å apart to generate the observed FRET efficiency. Given the relationship of residue 204 to the CRO (Figure 1), neither distances are feasible in a covalently linked dimer. What is clear is that bringing two different FPs in close proximity so promoting inter‐FP interactions does influence apparent FRET, which results in an observed FRET efficiency that generates an overestimation of the distance between the pair. Thus, while FRET is routinely assessed using differences in donor and acceptor fluorescence intensities based on the general idea presented in Equation (3), it does bring into question whether such analysis is appropriate in all cases.

To understand the impact of the observed effects for typical in vitro and in vivo investigations into protein interactions using FRET capable FPs fused to proteins of interest, we then assessed FRET between sfGFP and Venus in a non‐covalently linked complex. The FPs were fused to two known interacting proteins: Bcl3 and P50.^[^ ^28^ ^]^ The first construct comprised Bcl3 fused at the N‐terminal to sfGFP with no artificial linker (sfGFP‐Bcl3; Figure S8a, Supporting Information). The second construct comprised Venus fused to the C‐terminal of P50 via a largely unstructured 10 amino acid sequence (including a GSS artificial linker sequence) to allow for flexibility (Figure S8a, Supporting Information). The proteins were produced and purified as the complex, as outlined in the Supporting Methods and Figure S8b, Supporting Information. In this configuration the FPs will be positioned at the same end in the complex potentially allowing the FPs to interact in manner similar to that for GFVen^204^ (Figure S8c, Supporting Information). FRET was observed on excitation at 420 nm, with the major emission peak equivalent to Venus (*λ*_EM_, 529 nm; Figure 4d). The equivalent emission for P50‐Venus alone was much lower. The overall FRET efficiency was 85%, similar to that observed for the covalent GFVen^204x2^ dimer. This in turn raises the question concerning which system (closely associated or free rotating) is being sampled given the similarity of FRET efficiency with the covalently linked dimer. However, even using standard *R*_0_ of 55 Å, the two FP chromophores will be ≈41 Å apart and thus relatively close to each other in space so increasing the likelihood of association (and the accompanying effects on fluorescence as noted above), despite the relatively long flexible sequence that comprises the P50 construct. Alternatively, if a preferential TDM alignment between the CROs occurs, then *R*_0_ would be closer to 75 Å resulting in an interchromophore distance of ≈57 Å, which rudimentary structural modeling suggests is a more likely distance given that the Venus element is likely to sample greater dynamical freedom (Figure S8c, Supporting Information). Thus, using fluorescence intensity changes to measure FRET with no understanding of positional relationship of FP pairs may not fully reflect the molecular events in protein complexes.

### 2.4 Association of Green and Red Fluorescent Proteins

We next linked sfGFP with a DsRed‐derived monomeric protein, mCherry.^[^ ^29^ ^]^ Green fluorescent proteins can be used as an FRET partner with mCherry^[^ ^14^, ^30^ ^]^ with an estimated *J* coupling of 1.8 × 10^15^ M^−1^ cm^−1^ nm^4^ (FPbase FRET tool; www.fpbase.org/fret/).^[^ ^26^ ^]^ The sfGFP^204SCO^ variant was reacted with mCherry containing azF at the structurally equivalent position, residue 198 (Figure 4e). Molecular docking suggested the two proteins can associate at the interface between residues 204^sfGFP^ and 198^mCherry^ (Figure 4e), with covalent coupling via SPAAC subsequently proved by SDS PAGE (Figure S9, Supporting Information). Incorporation of azF at residue 198 in mCherry had little effect on the spectral properties of the monomer with a similar molar absorbance and brightness to the wt mCherry (69 000 M^−1^ cm^−1^ with a quantum yield of 24% compared to 72 000 M^−1^ cm^−1^ for wt mCherry with quantum yield of 22% at 587 nm; Figure 4g; Figure S9c,d, Table S1, Supporting Information).

The purified dimer, termed GFCh^x2^ did not appear to display any significant FRET on excitation at 490 nm (Figure 4f). Indeed, very little observable fluorescence can be attributed to mCherry in the dimer even on excitation at 585 nm (Figure 4h), which is confirmed visually through general UV excitation (Figure S9b, Supporting Information). The mCherry associated peak at ≈585 nm is reduced in terms of molar absorbance compared to the mCherry^198azF^ monomer. As with other dimeric forms (vide supra and ref. ^[^ ^11^ ^]^), the sfGFP molar absorbance increased above the simple addition of the two monomeric forms (≈16 000 M^−1^ cm^−1^ taking into account the contribution from the mCherry chromophore) confirming the role of dimerization via residue 204 in enhancing sfGFP function.

Given that donor fluorescence from the sfGFP component is prevalent in GFCh^x2^ (Figure 4f), it is unlikely that non‐radiative energy release from mCherry is the main cause of fluorescence loss as quenching of sfGFP fluorescent signal would also be observed. Data suggests that it is the interaction and subsequent conformation changes on interfacing with sfGFP that is responsible for loss of mCherry associated fluorescence, through altering the intrinsic properties of the chromophore. The reduction in the molar of absorbance of the mCherry component provide some credence to this idea given that molar absorbance is generally enhanced in dimers comprised solely of *A. victoria* derived FPs.^[^ ^11^ ^]^ One possibility is that dimerization is shifting the mCherry chromophore to a chemically modified, non‐functional conformation, akin to that observed for reduced versions of the chromophore^[^ ^31^ ^]^ or for dark state versions of photosensitive versions of the protein (e.g., PAmCherry).^[^ ^32^ ^]^ However, the presence of a clear, albeit less intense mCherry absorbance peak in GFCh^x2^ suggest that a significant proportion of the chromophore has not been chemically altered. Thus, it is more likely that chromophore is switched and then trapped in an alternative non‐fluorescent conformation (e.g., *cis* to *trans* isomerization),^[^ ^33^ ^]^ as occurs in the mCherry photoswitching variant rsCherry.^[^ ^34^ ^]^ Given the proximity of the mCherry chromophore to the dimer interface, dimerization may alter the conformation surrounding residues so promoting the *trans* over the *cis*.^[^ ^33^ ^]^ Such phenomena would open up the opportunity for engineering FP probes capable of photoswitching upon the interaction of their fusion protein partners. Reaction of mCherry^198azF^ with the SCO‐K ncAA alone does not appear to affect fluorescence suggesting the covalent linkage per se is not responsible (Figure S9e, Supporting Information). Attachment with the bulker azide containing Cy3 dye also does not result in loss of fluorescence, with FRET observed as expected (Figure S9f, Supporting Information). Thus, placing the two FPs in close proximity to promote their interaction is the likely cause of the loss in fluorescence. As donor fluorescence is still observed, in a classical ensemble FRET experiment this could be interpreted as the two target proteins not interacting when the opposite may in fact be the case. As pointed out earlier, basing FRET efficiency solely on intensity changes may thus not be appropriate in all cases.

### 2.5 Comparison with Alternative sfGFP Dimer sfGFP^148x2^

**Figure 5:**
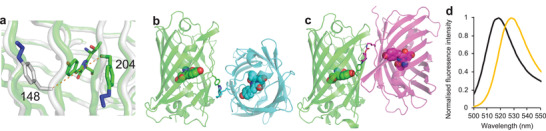
Comparison of sfGFP^204x2^ structure with sfGFP^148x2^. a) Location of the azF moiety at residue 148 (grey) or 204 (green). Structure of b) sfGFP^204x2^ and c) sfGFP^148x2^. Each structure is orientated identically with respect to the azF containing ncAA (colored green) to highlight the relative differences in monomer arrangements. d) Normalized emission on excitation at 490 nm for GFVen^148^ (black) and wt Venus (gold).

The structure of another click‐linked dimer joined via residue 148 (termed sfGFP^148x2^) has recently been reported.^[^ ^11^ ^]^ Dimerization effectively switched sfGFP^148x2^ on, with the dimer displaying improved function compared to both monomers and the original wild type sfGFP (sfGFP^WT^). We used the structure of the sfGFP^148x2^ dimer to calculate *κ* ^2^ as a representative alternative CRO arrangement. This will in turn allow us to investigate how different configurations of one monomer to the other affect dipole alignments and hence FRET. Residue 204 lies close to 148 on the adjacent *β*‐strand (**Figure** **5**a) but they adopt very different sidechain and thus monomer arrangements in their crystal structures (Figure 5b,c). In contrast to sfGFP^204x2^, the triazole link in sfGFP^148x2^ forms the extended anti form that is re‐enforced with both polar and hydrophobic interactions between the monomers generating a quasi‐symmetrical “head‐to‐tail” arrangement of the monomers. The result of such a configurational change between the two monomers units results in the relative positioning of the CROs being very different (compare Figures 5b and 5c).

Using the same approach as for GFVen^204^, we calculated *κ* ^2^ values for a model of GFVen^148^ based on the sfGFP^148x2^ structure. It should be noted that unlike linkage through residues 204 (or 198 in mCherry), covalent coupling via residue 148 was designed to instigate a functional change through synergistic conformation events.^[^ ^11^ ^]^ However, it does allow us to assess how changing the orientation and inter‐unit interactions of one monomer to another along a quasi‐similar interface region alters dipole arrangements. The calculated *κ* ^2^ was 3.79, even closer to the maximal value of 4 than sfGFP^204x2^. While this would suggest a longer *R*_0_ distance than GFVen^204^, the inherent function of the GFVen^148^ dimer system makes calculating *R*_0_ problematic; the donor, sfGFP^SCO148^, is essentially switched off in monomeric state and only becomes activated on dimerization. However, the main effect that will influence any FRET analysis is the shift in *λ*_EM_, which is blue shifted by 10 nm in the GFVen^148^ dimer compared to Venus^WT^ (Figure 5d) when excited at a wavelength corresponding to sfGFP. If single wavelength readings are taken with 530 nm assumed to be the Venus emission maximum, fluorescence emission would be underestimated by up to 35% so impacting on perceived FRET efficiency. While residue 204 is more applicable in terms of understanding association and FRET due to the non‐perturbative nature of the initial mutations, sfGFP^148x2^ and GFVen^148^ still act as good examples of how association is once again having a significant effect on the spectral properties. It also demonstrates that FP dimerization are not restricted to a defined interaction configuration but that different inter‐FP orientations are available.

## 3 Conclusion

**Figure 6:**
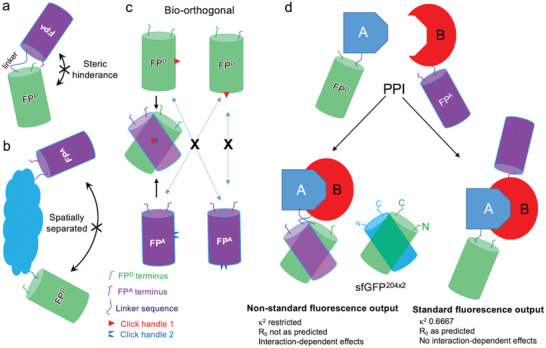
Schematic outline of assessing FP pairs. FP^D^ and FP^A^ refer to nominal FP donor and acceptor for FRET. a) Model system where short linkers generating a tandem FP pair. Linkers may be too short to allow full freedom to sample interactions, especially side‐on interactions. So, there is a low likelihood of FP interaction. b) Model system whereby FP pairs are bridged by a protein domain or whole protein. If the termini of the bridging protein are at opposite ends then the two FPs will be spatially separated so cannot interact. c) Bio‐orthogonal click chemistry approach whereby one FP has one type of chemistry (e.g., azide) and the second a mutually reactive handle (shown as red triangle and blue inverted triangle). Only FPs with mutually compatible interfaces will react and so stabilize the interaction. If the interface is not compatible the FPs will not click together. Broader FP–FP interface regions can be sampled through this approach. d) Protein–protein interaction (PPI) system. Two scenarios are envisaged. The first is that on interaction of protein A and B, the FPs are brought in close proximity to each other promoting association, which may in turn lead to non‐standard fluorescence properties. In the second scenario, protein A and B interact but the FPs remain spatially separate so displaying more classical fluorescence behavior.

Our ability to construct dimers of FPs coupled with structural analysis has allowed us to look at how association can influence two of their key functions: inherent electronic excitation/light emission and communication through energy transfer. With regards to the latter, we can use structural information to predict dipole alignments of two CROs, which is critical to FRET through defining *κ* ^2^. In our case, the arbitrary 0.6667 for the *κ* ^2^ value provides a significant underestimate of the predicted values that impacts on *R*_0_. There have been several studies to date that measure FRET in constructs whereby FPs are coupled via linker sequences or whole protein domains. However, by linking two FPs together they can no longer freely interact with each other due to, for example, steric hindrance (e.g., when using linker sequences)^[^ ^13^ ^]^ or spatially forced apart (e.g., when linked to via a single whole protein),^[^ ^12^ ^]^ schematically outlined in **Figure** **6**. Our use of bio‐orthogonal chemistry allows broader sampling and stabilization of mutually compatible FP interfaces (Figure 6c); non‐compatible FP surfaces do not form covalent bonds so the interface will not persist.^[^ ^11^ ^]^ The most powerful use of FRET is monitoring protein–protein interactions whereby the FPs are fused to separate proteins. It can be argued that most FP fusions will not associate in most FRET experiments. However, as FPs will be attached to partner protein that normally associate, and if the two FPs are in close proximity they may well align or even interact in preferential arrangements (Figure 6d), which can define dipole alignment and influence inherent FP function. Naïve docking of FPs along with empirical evidence highlights FPs tendency to oligomerize, which will be enhanced by local high concentrations. Promotion of FP interactions can then potentially impact on calculated FRET efficiencies, critical for reporting of protein–protein interactions, using commonly used fluorescence intensities approaches related to Equation (3). Closer attention to the initial construction of FP fusion protein to prevent such interactions should help address this issue (Figure 6d), which in turn will allow standard approaches to FRET analysis to be applied in a more meaningful manner. It is thus clear from our work that by placing FPs in close proximity so promoting physical interaction can result in changes in the expected fluorescence behavior.

## 4 Experimental Section

##### Protein Production

The monomeric sfGFP^204azF^ and sfGFP^204SCO^ proteins and the sfGFP^204x2^ dimer were produced as described previously.^[^ ^11^ ^]^ The WT mCherry, mCherry^198azF^, sfGFP‐Bcl3, and P50‐Venus proteins were produced as outlined in the section Methods, Supporting Information.

##### Protein Dimerization and Conjugation

The procedures for generating sfGFP homodimers and sfGFP‐Venus heterodimers have been reported previously.^[^ ^11^ ^]^ Generation of the sfGFP‐mCherry dimers was performed as follows. The sfGFP^204SCO^ and mCherry^198azF^ were mixed at an equimolar concentration (50 µm, 50 mm Tris‐HCl pH 8.0) and left at room temperature for ≈16 h. Dimers were purified by size exclusion chromatography (Superdex 200, 16/600). Protein dimerization and separation was also monitored by SDS PAGE gel. Conjugation with non‐proteinaceous molecules is described in the section Methods, Supporting Information.

##### Steady State Absorbance and Fluorescence Analysis

Spectrophotometry and fluorescence were performed essentially as described previously for sfGFP monomers, dimers and Bcl3 fusion, Venus monomers, P50 fusions, and sfGFP–Venus hybrid dimers.^[^ ^11^ ^]^ Analysis of variants involving mCherry followed a similar analysis procedure, using proteins concentration of 5 µm in 50 mm Tris‐HCl pH 8.0. Absorbance spectra were recorded on a Cary Win UV, using a 300 nm min^−1^ scan rate at 1 nm intervals. Absorbance at *λ*_max_ for each variant, was used to determine the molar extinction coefficients (*ε*) for each variant, using the Beer–Lambert equation and measured protein concentrations. Emission spectra were collected on a Cary Varian fluorimeter at a scan rate of 60 nm min^−1^ and 1 nm intervals. Emission and excitation slit widths were set to 10 nm and a detector voltage of low. Samples were excited at 5 nm from 460 nm to 590 nm as stated in the main text and emission was scanned from the excitation wavelength to 800 nm. *J* coupling constants (*J*[*λ*]) were calculated using either available parameters on FPbase^[^ ^26^ ^]^ via the FRET tool or calculated from experimental data using a|e software (http://www.fluortools.com/software/ae). FRET efficiency was calculated using Equation (3). To determine the contribution of donor fluorescence in dimer constructs, dimer emission spectra where deconvoluted by subtracting the monomeric acceptor emission spectra so as to calculate residual emission of the donor.

##### Single Molecule Fluorescence

Measurement and analysis of single molecule sfGFP^204x2^ fluorescence by total internal resonance fluorescence microscopy was performed as described previously.^[^ ^11^ ^]^

##### Structure Determination of sfGFP^204x2^

The sfGFP^204x2^ dimer variant was concentrated in 50 mm Tris pH 8.0 to a final concentration of 10 mg mL^−1^, and used to set up vapor diffusion crystal trays. A JBScreen membrane (Jena Bioscience, Germany) was used initially to facilitate crystal growth, where large green crystals grew in a multitude of buffer conditions. Large green crystals grew in 20% polyethylene glycol w/v, 100 mm HEPES, which were harvested and transferred to mother liquor supplemented with 13% w/v PEG 200 as a cryo‐protectant, and vitrified in liquid nitrogen. X‐ray diffraction data was collected at the Diamond light source, Harwell, UK (beamline I02). Structure refinement was performed using the CCP4 program suite.^[^ ^35^ ^]^ The structure was solved initially using the molecular replacement program PHASER,^[^ ^36^ ^]^ with wt sfGFP (PDB accession 2B3P) used as a model. Structures were manually adjusted using with COOT,^[^ ^37^ ^]^ and refined with TLS restrained refinement using REFMAC.^[^ ^38^ ^]^ The final coordinates were deposited in the Protein Data Bank (PDB) under accession code 5NI3.

##### Kappa^2^ Calculation

The dipole orientation factor, *κ* ^2^, was calculated using an approach as described previously.^[^ ^39^ ^]^ The model structure of GFVen^204^ dimer was built by overlapping the WT structure of Venus (1MYW) onto the sfGFP^204azF^ component of sfGFP^204x2^ structure. While Venus and sfGFP used here had 15 amino acid differences in the core *β*‐barrel structure only Ala206 in sfGFP^SCO204^ and Val206 in Venus contributed to the domain interface. Residue 204 was then replaced with azF and linked to SCO using the PyMOL bond building tool. The GFVen^204^ model overlaid with sfGFP^204x2^ is shown in Figure S4, Supporting Information. Using previously established approaches,^[^ ^24^, ^39^ ^]^ the model structure *κ* ^2^ was calculated as outlined in Equation (4) using the distance between the centers of the donor and acceptor dyes (*r*_da_), and the orientations of the transition dipole moments of the donor ($\vec{d}$), and the acceptor ($\vec{a}$).^[^ ^39^ ^]^ The angles are defined as shown in Figure S10, Supporting Information.

(4) $$\begin{array}{c} k^{2}={\left[\vec{d}\cdot \vec{a}-3\left(\vec{d}\cdot r_{\mathrm{da}}\right)\left(\vec{a}\cdot r_{\mathrm{da}}\right)\right]}^{2}=\;{\left(\cos\theta_{T}-3\cos\theta_{D}\cos\theta_{A}\right)}^{2} \end{array}$$

The atomic positions of CG2 and C2 of the chromophore, as shown in Figure S6b, Supporting Information, were used to the define the vector for the TDM for both Venus and sfGFP.^[^ ^24^ ^]^ The *κ* ^2^ was then used in Equation (1) together with available experimental to calculate *R*_0_ for FRET pairs.

## Conflict of Interest

The authors declare no conflict of interest.

1a) 
E. A.
Rodriguez
, 
R. E.
Campbell
, 
J. Y.
Lin
, 
M. Z.
Lin
, 
A.
Miyawaki
, 
A. E.
Palmer
, 
X.
Shu
, 
J.
Zhang
, 
R. Y.
Tsien
, Trends Biochem. Sci.
2017, 42, 111;2781494810.1016/j.tibs.2016.09.010PMC5272834b) 
S.
Duwe
, 
P.
Dedecker
, Curr. Opin. Biotechnol.
2019, 58, 183.3117061010.1016/j.copbio.2019.04.0062a) 
N. C.
Shaner
, 
P. A.
Steinbach
, 
R. Y.
Tsien
, Nat. Methods
2005, 2, 905;1629947510.1038/nmeth819b) 
R. H.
Newman
, 
M. D.
Fosbrink
, 
J.
Zhang
, Chem. Rev.
2011, 111, 3614;2145651210.1021/cr100002uPMC3092831c) 
A.
Ibraheem
, 
R. E.
Campbell
, Curr. Opin. Chem. Biol.
2010, 14, 30.1991345310.1016/j.cbpa.2009.09.0333

A.
Miyawaki
, 
D. M.
Shcherbakova
, 
V. V.
Verkhusha
, Curr. Opin. Struct. Biol.
2012, 22, 679.2300003110.1016/j.sbi.2012.09.002PMC37372444

R. Y.
Tsien
, Annu. Rev. Biochem.
1998, 67, 509.975949610.1146/annurev.biochem.67.1.5095

M. V.
Matz
, 
A. F.
Fradkov
, 
Y. A.
Labas
, 
A. P.
Savitsky
, 
A. G.
Zaraisky
, 
M. L.
Markelov
, 
S. A.
Lukyanov
, Nat. Biotechnol.
1999, 17, 969.1050469610.1038/136576

P. J.
Cranfill
, 
B. R.
Sell
, 
M. A.
Baird
, 
J. R.
Allen
, 
Z.
Lavagnino
, 
H. M.
de Gruiter
, 
G. J.
Kremers
, 
M. W.
Davidson
, 
A.
Ustione
, 
D. W.
Piston
, Nat. Methods
2016, 13, 557.2724025710.1038/nmeth.3891PMC49273527

D. A.
Zacharias
, 
J. D.
Violin
, 
A. C.
Newton
, 
R. Y.
Tsien
, Science
2002, 296, 913.1198857610.1126/science.10685398

T.
Förster
, Ann. Phys.
1948, 437, 55.9

B.
Hellenkamp
, 
S.
Schmid
, 
O.
Doroshenko
, 
O.
Opanasyuk
, 
R.
Kuhnemuth
, 
S. R.
Adariani
, 
B.
Ambrose
, 
M.
Aznauryan
, 
A.
Barth
, 
V.
Birkedal
, 
M. E.
Bowen
, 
H.
Chen
, 
T.
Cordes
, 
T.
Eilert
, 
C.
Fijen
, 
C.
Gebhardt
, 
M.
Gotz
, 
G.
Gouridis
, 
E.
Gratton
, 
T.
Ha
, 
P.
Hao
, 
C. A.
Hanke
, 
A.
Hartmann
, 
J.
Hendrix
, 
L. L.
Hildebrandt
, 
V.
Hirschfeld
, 
J.
Hohlbein
, 
B.
Hua
, 
C. G.
Hubner
, 
E.
Kallis
, et al., Nat. Methods
2018, 15, 669.3017125210.1038/s41592-018-0085-0PMC612174210a) 
S. C.
Alford
, 
Y.
Ding
, 
T.
Simmen
, 
R. E.
Campbell
, ACS Synth. Biol.
2012, 1, 569;2365627810.1021/sb300050jPMC3653836b) 
X. X.
Zhou
, 
H. K.
Chung
, 
A. J.
Lam
, 
M. Z.
Lin
, Science
2012, 338, 810.2313933510.1126/science.1226854PMC370205711

H. L.
Worthy
, 
H. S.
Auhim
, 
W. D.
Jamieson
, 
J. R.
Pope
, 
A.
Wall
, 
R.
Batchelor
, 
R. L.
Johnson
, 
D. W.
Watkins
, 
P.
Rizkallah
, 
O. K.
Castell
, 
D. D.
Jones
, Commun. Chem.
2019, 2, 83.12

P.
Trigo‐Mourino
, 
T.
Thestrup
, 
O.
Griesbeck
, 
C.
Griesinger
, 
S.
Becker
, Sci. Adv.
2019, 5, eaaw4988.3145708810.1126/sciadv.aaw4988PMC668572413

M. D.
Wiens
, 
Y.
Shen
, 
X.
Li
, 
M. A.
Salem
, 
N.
Smisdom
, 
W.
Zhang
, 
A.
Brown
, 
R. E.
Campbell
, ChemBioChem
2016, 17, 2361.2778139410.1002/cbic.20160049214

A. J.
Lam
, 
F.
St‐Pierre
, 
Y.
Gong
, 
J. D.
Marshall
, 
P. J.
Cranfill
, 
M. A.
Baird
, 
M. R.
McKeown
, 
J.
Wiedenmann
, 
M. W.
Davidson
, 
M. J.
Schnitzer
, 
R. Y.
Tsien
, 
M. Z.
Lin
, Nat. Methods
2012, 9, 1005.2296124510.1038/nmeth.2171PMC346111315

L. H.
Lindenburg
, 
A. M.
Hessels
, 
E. H.
Ebberink
, 
R.
Arts
, 
M.
Merkx
, ACS Chem. Biol.
2013, 8, 2133.2396215610.1021/cb400427bPMC382608316

M.
Mastop
, 
D. S.
Bindels
, 
N. C.
Shaner
, 
M.
Postma
, 
T. W. J.
Gadella
Jr.
, 
J.
Goedhart
, Sci. Rep.
2017, 7, 11999.2893189810.1038/s41598-017-12212-xPMC560732917

S. C.
Reddington
, 
P. J.
Rizkallah
, 
P. D.
Watson
, 
R.
Pearson
, 
E. M.
Tippmann
, 
D. D.
Jones
, Angew. Chem., Int. Ed.
2013, 52, 5974.10.1002/anie.2013014902362047218

S. C.
Reddington
, 
E. M.
Tippmann
, 
D. D.
Jones
, Chem. Commun.
2012, 48, 8419.10.1039/c2cc31887c2280145419

J. A.
Arpino
, 
P. J.
Rizkallah
, 
D. D.
Jones
, PLoS One
2012, 7, e47132.2307755510.1371/journal.pone.0047132PMC347305620

E. K.
Bomati
, 
J. E.
Haley
, 
J. P.
Noel
, 
D. D.
Deheyn
, Sci. Rep.
2014, 4, 5469.2496892110.1038/srep05469PMC407312121a) 
K.
Brejc
, 
T. K.
Sixma
, 
P. A.
Kitts
, 
S. R.
Kain
, 
R. Y.
Tsien
, 
M.
Ormo
, 
S. J.
Remington
, Proc. Natl. Acad. Sci. U. S. A.
1997, 94, 2306;912219010.1073/pnas.94.6.2306PMC20083b) 
M. H.
Seifert
, 
D.
Ksiazek
, 
M. K.
Azim
, 
P.
Smialowski
, 
N.
Budisa
, 
T. A.
Holak
, J. Am. Chem. Soc.
2002, 124, 7932.1209533710.1021/ja025772522a) 
A. M.
Hartley
, 
H. L.
Worthy
, 
S. C.
Reddington
, 
P. J.
Rizkallah
, 
D. D.
Jones
, Chem. Sci.
2016, 7, 6484;2845110610.1039/c6sc00944aPMC5355941b) 
A.
Shinobu
, 
N.
Agmon
, J. Chem. Theory Comput.
2017, 13, 353;2806876810.1021/acs.jctc.6b00939c) 
A.
Shinobu
, 
G. J.
Palm
, 
A. J.
Schierbeek
, 
N.
Agmon
, J. Am. Chem. Soc.
2010, 132, 11093.2069867510.1021/ja101065223

D.
Kozakov
, 
D. R.
Hall
, 
B.
Xia
, 
K. A.
Porter
, 
D.
Padhorny
, 
C.
Yueh
, 
D.
Beglov
, 
S.
Vajda
, Nat. Protoc.
2017, 12, 255.2807987910.1038/nprot.2016.169PMC554022924

T.
Ansbacher
, 
H. K.
Srivastava
, 
T.
Stein
, 
R.
Baer
, 
M.
Merkx
, 
A.
Shurki
, Phys. Chem. Chem. Phys.
2012, 14, 4109.2233109910.1039/c2cp23351g25

A.
Kyrychenko
, 
M. V.
Rodnin
, 
C.
Ghatak
, 
A. S.
Ladokhin
, Anal. Biochem.
2017, 522, 1.2810816810.1016/j.ab.2017.01.01126

T. J.
Lambert
, Nat. Methods
2019, 16, 277.3088641210.1038/s41592-019-0352-827

B. T.
Andrews
, 
A. R.
Schoenfish
, 
M.
Roy
, 
G.
Waldo
, 
P. A.
Jennings
, J. Mol. Biol.
2007, 373, 476.1782271410.1016/j.jmb.2007.07.071PMC269565628

B.
Manavalan
, 
S.
Basith
, 
Y. M.
Choi
, 
G.
Lee
, 
S.
Choi
, PLoS One
2010, 5, e15782.2120342210.1371/journal.pone.0015782PMC300974729a) 
X.
Shu
, 
N. C.
Shaner
, 
C. A.
Yarbrough
, 
R. Y.
Tsien
, 
S. J.
Remington
, Biochemistry
2006, 45, 9639;1689316510.1021/bi060773lb) 
N. C.
Shaner
, 
R. E.
Campbell
, 
P. A.
Steinbach
, 
B. N.
Giepmans
, 
A. E.
Palmer
, 
R. Y.
Tsien
, Nat. Biotechnol.
2004, 22, 1567.1555804710.1038/nbt103730a) 
B. T.
Bajar
, 
E. S.
Wang
, 
S.
Zhang
, 
M. Z.
Lin
, 
J.
Chu
, Sensors
2016, 16, 1488;b) 
D.
Shcherbo
, 
E. A.
Souslova
, 
J.
Goedhart
, 
T. V.
Chepurnykh
, 
A.
Gaintzeva
, 
Shemiakina
II
, 
T. W.
Gadella
, 
S.
Lukyanov
, 
D. M.
Chudakov
, BMC Biotechnol.
2009, 9, 24;1932101010.1186/1472-6750-9-24PMC2678114c) 
G. N.
van der Krogt
, 
J.
Ogink
, 
B.
Ponsioen
, 
K.
Jalink
, PLoS One
2008, 3, e1916.1838268710.1371/journal.pone.0001916PMC227105331

B. M. C.
Cloin
, 
E.
de Zitter
, 
D.
Salas
, 
V.
Gielen
, 
G. E.
Folkers
, 
M.
Mikhaylova
, 
M.
Bergeler
, 
B.
Krajnik
, 
J.
Harvey
, 
C. C.
Hoogenraad
, 
L.
van Meervelt
, 
P.
Dedecker
, 
L. C.
Kapitein
, Proc. Natl. Acad. Sci. U. S. A.
2017, 114, 7013.2863028610.1073/pnas.1617280114PMC550258832

F. V.
Subach
, 
V. N.
Malashkevich
, 
W. D.
Zencheck
, 
H.
Xiao
, 
G. S.
Filonov
, 
S. C.
Almo
, 
V. V.
Verkhusha
, Proc. Natl. Acad. Sci. U. S. A.
2009, 106, 21097.1993403610.1073/pnas.0909204106PMC279549433

F. V.
Subach
, 
V. V.
Verkhusha
, Chem. Rev.
2012, 112, 4308.2255923210.1021/cr2001965PMC339491034

A. C.
Stiel
, 
M.
Andresen
, 
H.
Bock
, 
M.
Hilbert
, 
J.
Schilde
, 
A.
Schonle
, 
C.
Eggeling
, 
A.
Egner
, 
S. W.
Hell
, 
S.
Jakobs
, Biophys. J.
2008, 95, 2989.1865822110.1529/biophysj.108.130146PMC252727835
Collaborative Computational Project Number 4
, Acta Crystallogr., Sect. D: Biol. Crystallogr.
1994, 50, 760.1529937436

A. J.
McCoy
, 
R. W.
Grosse‐Kunstleve
, 
P. D.
Adams
, 
M. D.
Winn
, 
L. C.
Storoni
, 
R. J.
Read
, J. Appl. Crystallogr.
2007, 40, 658.1946184010.1107/S0021889807021206PMC248347237

P.
Emsley
, 
K.
Cowtan
, Acta Crystallogr., Sect. D: Biol. Crystallogr.
2004, 60, 2126.1557276510.1107/S090744490401915838

G. N.
Murshudov
, 
A. A.
Vagin
, 
E. J.
Dodson
, Acta Crystallogr., Sect. D: Biol. Crystallogr.
1997, 53, 240.1529992610.1107/S090744499601225539

D. D.
Fernandes
, 
J.
Bamrah
, 
S.
Kailasam
, 
G. W.
Gomes
, 
Y.
Li
, 
H. J.
Wieden
, 
C. C.
Gradinaru
, Sci. Rep.
2017, 7, 13063.2902619510.1038/s41598-017-13427-8PMC5638890

## Supporting information

Supporting Information
