# Supplementary material for: Association of Fluorescent Protein Pairs and Its Significant Impact on Fluorescence and Energy Transfer
Source: Adv Sci (Weinh). 2020 Nov 23;8(1):2003167. doi: 10.1002/advs.202003167 (PMC7788595; doi:10.1002/advs.202003167)
Supplement: Supplementary file 1 — Supporting Information [file ADVS-8-2003167-s001.pdf]

© 2020 Wiley-VCH GmbH

ADVANCED SCIENCE
Open Access

Supporting Information

for *Adv. Sci.,* DOI: 10.1002/advs.202003167

Association of Fluorescent Protein Pairs and Its Significant Impact on Fluorescence and Energy Transfer

*Jacob R. Pope, Rachel L. Johnson, W. David Jamieson, Harley L. Worthy, Senthilkumar Kailasam, Rochelle D. Ahmed, Ismail Taban, Husam Sabah Auhim, Daniel W. Watkins, Pierre J. Rizkallah, Oliver K. Castell, and D. Dafydd Jones**

Supporting information for "Association of fluorescent protein pairs and it's significant impact on fluorescence and energy transfer"

Jacob R. Pope[1], Rachel L. Johnson[1], W. David Jamieson[2], Harley L Worthy[1], Senthilkumar Kailasam[5,6], Rochelle D. Ahmed[1], Ismail Taban[1], Husam Sabah Auhim[1], Daniel W. Watkins[1], Pierre Rizkallah[3], Oliver Castell[2], D. Dafydd Jones[1]*.

1. Molecular Biosciences, School of Biosciences, Cardiff University, Cardiff, UK.
2. School of Pharmacy, Cardiff University, Cardiff, UK.
3. School of Medicine, Cardiff University, Cardiff, UK.
4. Department of Biology, College of Science, Baghdad University, Baghdad, Iraq.
5. McGill University and Genome Quebec Innovation Centre, Montreal, QC, Canada,
6. Department of Human Genetics, McGill University, Montreal, QC, Canada

* Corresponding author. D. Dafydd Jones, Molecular Biosciences Division, School of Biosciences, Cardiff University, Cardiff, UK. CF10 3AT. Email: jonesdd@cardiff.ac.uk. Tel: +44 29 20874290.

Supporting Methods
Supporting Tables S1-S2
Supporting Figures S1-S10

Gene sequences

sfGFP-Bcl3

ATGCATCATCATCATCATCATGGTAGCAGCGTTAGCAAAGGTGAAGAACTGTTTACCGGCGTTGTGCC
GATTCTGGTGGAACTGGATGGTGATGTGAATGGCCATAAATTTAGCGTTCGTGGCGAAGGCGAAGGTG
ATGCGACCAACGGTAAACTGACCCTGAAATTTATTTGCACCACCGGTAAACTGCCGGTTCCGTGGCCG
ACCCTGGTGACCACCCTGACCTATGGCGTTCAGTGCTTTAGCCGCTATCCGGATCATATGAAACGCCA
TGATTTCTTTAAAAGCGCGATGCCGGAAGGCTATGTGCAGGAACGTACCATTAGCTTCAAAGATGATG
GCACCTATAAAACCCGTGCGGAAGTTAAATTTGAAGGCGATACCCTGGTGAACCGCATTGAACTGAAA
GGTATTGATTTTAAAGAAGATGGCAACATTCTGGGTCATAAACTGGAATATAATTTCAACAGCCATAA
TGTGTATATTACCGCCGATAAACAGAAAAATGGCATCAAAGCGAACTTTAAAATCCGTCACAACGTGG
AAGATGGTAGCGTGCAGCTGGCGGATCATTATCAGCAGAATACCCCGATTGGTGATGGCCCGGTGCTG
CTGCCGGATAATCATTATCTGAGCACCCAGAGCGTTCTGAGCAAAGATCCGAATGAAAAACGTGATCA
TATGGTGCTGCTGGAATTTGTTACCGCCGCGGGCGCGACCCGCGCGGATGAGGATGGCGATACTCCGT
TACACATTGCGGTGGTCCAAGGCAACTTGCCGGCTGTGCACCGTCTGGTTAATCTTTTCCAGCAGGGC
GGCCGTGAACTTGACATCTATAACAATTTGCGTCAAACCCCACTGCATCTGGCTGTTATTACCACCCT
GCCGAGCGTGGTTCGCTTATTGGTGACCGCAGGGGCATCGCCTATGGCACTGGACCGTCATGGCCAGA
CCGCTGCCCATCTTGCGTGCGAACATCGGAGCCCAACATGCTTGCGCGCGCTTCTGGACTCCGCGGCG
CCGGGTACCCTTGACTTAGAAGCGCGCAATTATGACGGTCTGACGGCATTACATGTTGCGGTGAACAC
CGAATGCCAGGAAACTGTGCAACTGCTGTTGGAACGTGGGGCCGATATCGATGCTGTAGATATCAAAT
CAGGTCGCTCGCCCTTAATTCACGCGGTTGAGAACAATAGCTTATCAATGGTACAACTGCTCCTGCAG
CATGGCGCGAATGTAAATGCGCAAATGTACTCCGGCAGTAGCGCGCTGCATTCGGCAAGCGGCCGTGG
TCTGCTTCCGCTCGTGCGCACTCTGGTCCGCTCTGGCGCCGACAGTTCCCTGAAAAATTGCCATAACG
ATACACCTCTGATGGTCGCGCGCAGCCGGCGCGTTATTGATATCTTACGCGGCAAAGCGACTCGCCCG
GCGAGCTAA

p50-Venus

ATGAGAGGATCTCACCATCACCATCACCATACGGATCCGGCCCTGAGGGCCGCAGATGGCCCATACCT
TCAAATATTAGAGCAACCTAAACAGAGAGGATTTCGTTTCCGTTATGTATGTGAAGGCCCATCCCATG
GTGGATTACCTGGTGCCTCTAGTGAAAAGAACAAGAAGTCTTACCCTCAGGTCAAAATCTGCAACTAT
GTGGGACCAGCAAAGGTTATTGTTCAGTTGGTCACAAATGGAAAAAATATCCACCTGCATGCCCACAG
CCTGGTGGGAAAACACTGTGAGGATGGGATCTGCACTGTAACTGCTGGACCCAAGGACATGGTGGTCG
GCTTCGCAAACCTGGGTATACTTCATGTGACAAAGAAAAAAGTATTTGAAACACTGGAAGCACGAATG
ACAGAGGCGTGTATAAGGGGCTATAATCCTGGACTCTTGGTGCACCCTGACCTTGCCTATTTGCAAGC
AGAAGGTGGAGGGGACCGGCAGCTGGGAGATCGGGAAAAAGAGCTAATCCGCCAAGCAGCTCTGCAGC
AGACCAAGGAGATGGACCTCAGCGTGGTGCGGCTCATGTTTACAGCTTTTCTTCCGGATAGCACTGGC
AGCTTCACAAGGCGCCTGGAACCCGTGGTATCAGACGCCATCTATGACAGTAAAGCCCCCAATGCATC
CAACTTGAAAATTGTAAGAATGGACAGGACAGCTGGATGTGTGACTGGAGGGGAGGAAATTTATCTTC
TTTGTGACAAAGTTCAGAAAGATGACATCCAGATTCGATTTTATGAAGAGGAAGAAAATGGTGGAGTC
TGGGAAGGATTTGGAGATTTTTCCCCCACAGATGTTCATAGACAATTTGCCATTGTCTTCAAAACTCC
AAAGTATAAAGATATTAATATTACAAAACCAGCCTCTGTGTTTGTCCAGCTTCGGAGGAAATCTGACT
TGGAAACCAGTGAACCAAAACCTTTCCTCTACTATCCTGAAATCAAAGATAAAGAAGAAGTGCAGAGG
AAACGTCAGAAGGGGAGCTCGCATATGATGGTGAGCAAGGGCGAGGAGCTGTTCACCGGGGTGGTGCC
CATCCTGGTCGAGCTGGACGGCGACGTAAACGGCCACAAGTTCAGCGTGTCCGGCGAGGGCGAGGGCG
ATGCCACCTACGGCAAGCTGACCCTGAAGCTGATCTGCACCACCGGCAAGCTGCCCGTGCCCTGGCCC
ACCCTCGTGACCACCCTGGGCTACGGCCTGCAGTGCTTCGCCCGCTACCCCGACCACATGAAGCAGCA
CGACTTCTTCAAGTCCGCCATGCCCGAAGGCTACGTCCAGGAGCGCACCATCTTCTTCAAGGACGACG
GCAACTACAAGACCCGCGCCGAGGTGAAGTTCGAGGGCGACACCCTGGTGAACCGCATCGAGCTGAAG
GGCATCGACTTCAAGGAGGACGGCAACATCCTGGGGCACAAGCTGGAGTACAACTACAACAGCCACAA
CGTCTATATCACCGCCGACAAGCAGAAGAACGGCATCAAGGCCAACTTCAAGATCCGCCACAACATCG
AGGACGGCGGCGTGCAGCTCGCCGACCACTACCAGCAGAACACCCCCATCGGCGACGGCCCCGTGCTG
CTGCCCGACAACCACTACCTGAGCTACCAGTCCGCCCTGAGCAAAGACCCCAACGAGAAGCGCGATCA
CATGGTCCTGCTGGAGTTCGTGACCGCCGCCGGGATCACTCTCGGCATGGACGAGCTGTACAAGTAA

Construction and production of Bcl3-sfGFP/Venus-P50 complex
The sfGFP-Bcl3 and p50-venus (see above for constructs) were cloned into pET11a and the pCA24N vectors, respectively. The two plasmids where co-transformed by electroporation into *E.coli* BL21 (DE3) (NEB), which where used to inoculate 1L flasks of 2xYT (Melford) supplemented with 100 μg/mL carbenicillin and 35 μg/mL chloramphenicol. Cultures were grown at 37 °C until an OD_{600} of 0.6 was achieved, and protein expression induced by addition of 0.4 mM IPTG. The cells where grown overnight at 27 °C. Cells were harvested and were lysed using a French press. The resulting lysate was clarified by centrifugation and loaded onto a 5 mL HisTrapHP^{TM} (GE Healthcare) column equilibrated in equilibration-wash buffer (20 mM HEPES, 300 mM NaCl, 10 mM imidazole, pH8.0) then allowed to drain by gravity. Bound uncomplexed protein (sfGFP-Bcl3 and p50-venus) were eluted by washing the column with 250 mM imidazole. Complex (Bcl3-sfGFP/p50-Venus) was eluted by washing the column with 500 mM imidazole. Samples containing the complex were then loaded onto a HiLoad^{TM} 26/600Superdex^{TM} S200 pg column equilibrated in 20 mM HEPES, 300 mM NaCl, pH8.0. Purity was checked via SDS-PAGE analysis. Fluorescence analysis was performed as outlined in the main manuscript.

Construction and production of mCherry variants. The gene encoding wt mCherry resident within the pBAD plasmid was used to generate the K198TAG mutation by whole plasmid site-directed mutagenesis (Forward primer 5'-CGGCGCCTACAACGTCAACATC**TAG**T-3' and reserve primer 5'-GGCAGCTGCACGGGCTTCTT-3') using Q5 polymerase (NEB, USA). *E. coli* Top10 cells were transformed and used to inoculate 1L flasks of LB media supplemented with 100 µg/mL ampicillin, 25 µg/mL tetracycline. To incorporate azF, cells were also co-transformed with pDULEcyanoRS and cultured in the presence of 0.1 mM azF. Cultures were grown for 1 hour at 37°C in a shaking incubator before expression was induced by addition of 0.1% of arabinose and incubated for 24 hours at 25°C. Cultures were kept in the dark until after dimerisation with SCO-K containing variants.

Cells were harvested via centrifugation and resuspended in 20 mL of 50 mM Tris-HCl pH8.0, 1 mM EDTA. The cells were lysed using a French press and the resulting lysate was clarified by centrifugation. Cell lysates were then loaded onto a 5 mL HisTrapHP™ (GE Healthcare) equilibrated in lysis buffer. Bound protein was eluted by washing the column in 250 mM Imidazole. Samples were then loaded onto a Superdex 75 column equilibrated in 50 mM Tris-HCl pH8.0 and purity was checked via SDS-PAGE analysis. Concentrations of monomer variants were determined using the Bio-RAD DC Protein Assay using wild type wt mCherry as a standard and correlated to the 280 nm absorbance. The quantum yield of mCherry^{198azF} was calculated as described previously (*1*) using WT mCherry as the reference sample.

Conjugation of mCherry with non-proteinaceous molecules. Conjugation of mCherry^{198azF} with the ncAA SCO-K was performed in 50 mM Tris-HCl pH 8.0) with 5 μM protein and 200 μM of ncAA. Samples were left for 4 hr at room temperature and analysed by absorbance and fluorescence spectroscopy. Conjugation of mCherry^{198azF} with Cy3 DBCO (Click Chemistry Tools, USA) was performed with equimolar concentrations of protein and dye (5 μM). The absorbance and fluorescence emission were recorded immediately after sample mixing. The sample was then left at room temperature overnight. Following overnight incubation, the

absorbance and fluorescence were performed again, and the reaction mix was run on SDS page gel.

Supporting Table S1. Spectral properties of Fluorescent protein variants.

Variant	λ_{max} (nm)	λ_{EM} (nm)	ε ($M^{-1}cm^{-1}$)	QY	Brightness
$sfGFP^{WT}$	485	511	49000[a]	0.75	36750
$sfGFP^{204SCO}$	485	511	39800	0.66	26268
$sfGFP^{204azF}$	485	511	51000	0.68	34680
$sfGFP^{204x2}$ [b]	490	513	160000	0.71	113600
$Venus^{WT}$ [c]	515	528	92200	0.65	59930
$Venus^{204azF}$	515	528	87600	0.42	36792
$GFVen^{204}$ [b]	492	530	102000	0.70	71400
	514	530	116800	0.60	70800
$mCherry^{WT}$ [d]	587	610	72000	0.22	15840
$mCherry^{198azF}$	587	610	69000	0.24	16560
$GFCh^{x2}$	488	511	60000	0.52	31200
	587	-	31000	-	-

[a] We have reported previously a significant shortfall in the molar absorbance coefficient we routinely calculate (here and (*2-4*)) and that published by Pedelacq et al (*5*). [b] Published previously by Worthy et al (*6*). [c] Published previously by Nagai et al (*7*). [d] Published previously by Sharner et al (*8*).

Supporting Table S2. Crystallographic statistics for $sfGFP^{204x2}$

	**GFP^{204x2}**
PDB ID	5NI3
Wavelength (Å)	0.979
Beamline	Diamond IO2
Space group	$P2_12_12_1$
a (Å)	96.700
b (Å)	98.020
c (Å)	102.640
Resolution range (Å)	57.17-1.28
Total reflections measured	1,840,570
Unique reflections	249,821
Completeness (%) (last shell)	99.9(99.8)
Multiplicity (last shell)	7.2 (7.1)
I/σ (last shell)	17.7 (1.35)
CC1/2	0.999 (0.512)
R(merge)[a] (%) (last shell)	4.5(1.68)
B(iso) from Wilson ($Å^2$)	15.12
B(iso) from refinement	23.7
Log Likelihood Coordinate rms	0.036
Non-H atoms	8,482
Solvent molecules	999
R-factor[b] (%)	15.8
R-free[c] (%)	17.3
Rmsd bond lengths (Å)	0.019
Rmsd bond angles (°)	2.143
Core region (%)	98.40
Allowed region (%)	1.13
Additionally allowed region (%)	0
Disallowed Region (%)	0.45
**Structural comparison**	
RMSD of azF monomer to sfGFP (Å)	0.193
RMSD of SCO monomer to sfGFP (Å)	0.165

Table S3. Calculated R_0 values based on available data

Data source	Donor	Acceptor	J ($M^{-1}cm^{-1}nm^{4}$)	QY (Donor)	R_0 (Å) κ^2=0.667	R_0 (Å) κ^2=3.59
FPbase[a]	sfGFP	Venus	3.03×10^{14}	0.65	55.75	73.81
Expt[b]	sfGFP	Venus	3.77×10^{14}	0.65	57.87	76.62
Expt[b]	$sfGFP^{204SOC}$	$Venus^{204azF}$	3.46×10^{14}	0.66	57.21	75.74

[a] available from the online fluorescent protein resource, FPbase (https://www.fpbase.org); [b] parameters experimentally determined by the authors.

Supporting Figures.

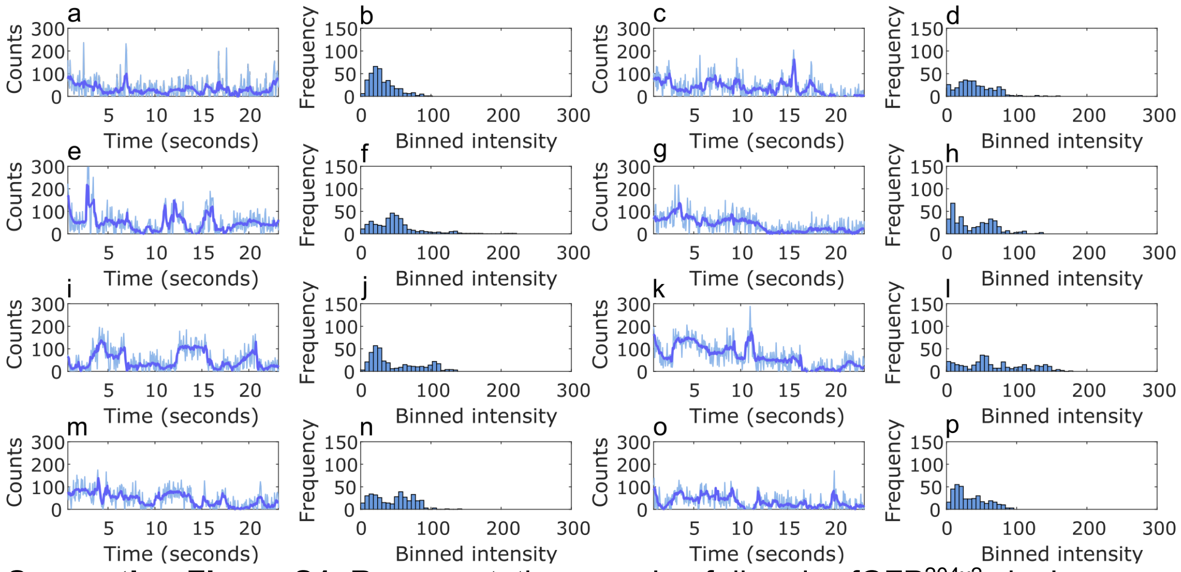

Supporting Figure S1. Representative sample of dimeric sfGFP^{204x2} single molecule time course traces (raw and Cheung-Kennedy filtered) coupled with paired intensity frequency histograms (generated from Cheung Kennedy filtered data) to the right of each trace.

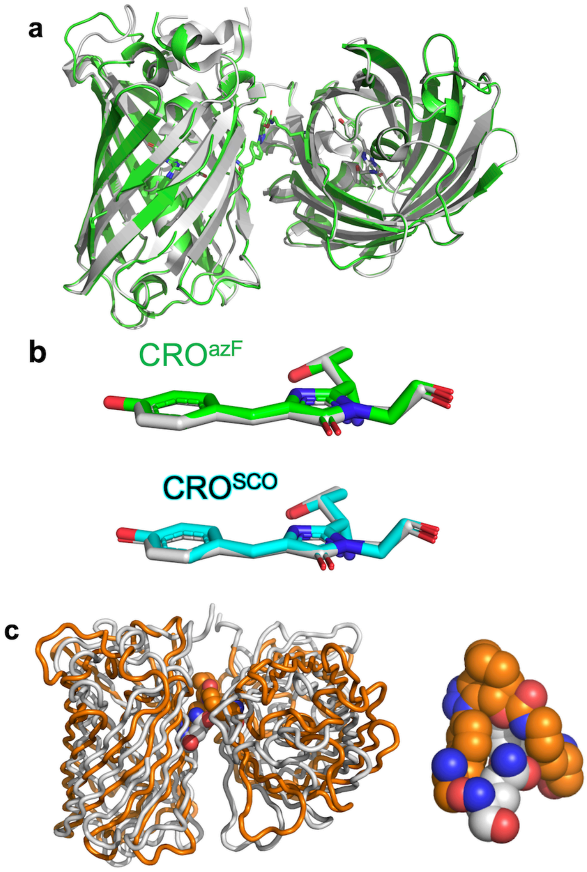

Supporting Figure S2. Structural analysis of sfGFP^{204x2}. (a) Overlay of the two observed dimers in 5NI3 with the WT sfGFP (grey). The Cα RMSD of WT sfGFP with the azF monomer is 0.193 Å and 0.165 Å with the SCO-K monomer. (b) Overlay

of chromophores from sfGFP^{WT} (grey), the sfGFP^{204azF} monomer unit (green) and the sfGFP^{204azF} monomer unit (cyan). (c) Overlay of best fitting model calculated previously (*6*) (coloured grey) and the determined structure of sfGFP^{204x2} (orange). Overlap of residues involved in the triazole crosslink are shown to the right as spheres. The Cα RMSD between the model and determined structure is 5.6 Å.

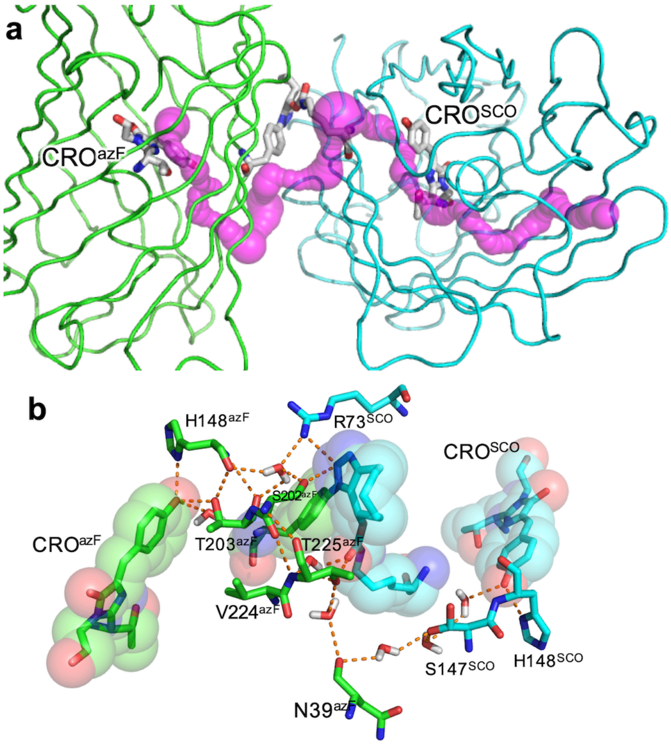

Supporting Figure S3. Long range tunnels and bond networks in sfGFP^{204x2}. (a) CAVER (*9*) analysis of sfGFP^{204x2}. The tunnel linking the CROs are coloured magenta. (b) Long range H-bond network linking the two CROs. The subscript to the residues denotes the monomer the residues are from. The sfGFP^{204azF} monomer is coloured green and the sfGFP^{204SCO} is coloured cyan. Additional information. Analysis of sfGFP^{204x2} using CAVER 21 suggests that a tunnel linking the two CROs is present (Figure 5a). The pathway extends beyond CRO^{SCO} to exit around residues 23, 54 and 136. An extended putative interaction polar network involving the SCO and triazole moieties, water and amino acids spans the cavity and links the two CROs. Compared to the GFP^{148x2}, the network is not as coherent or direct.

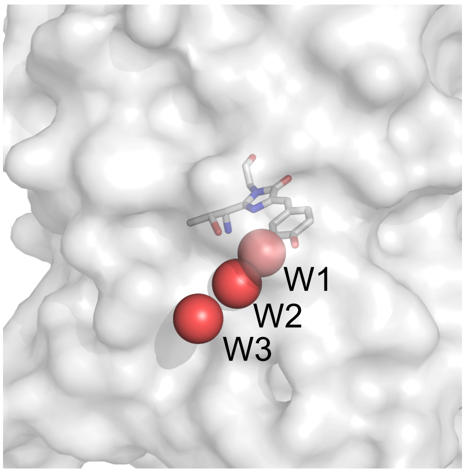

Supporting Figure S4. Water molecules (red spheres) closely associated with the CRO (grey sticks) in sfGFP^{WT} monomer.

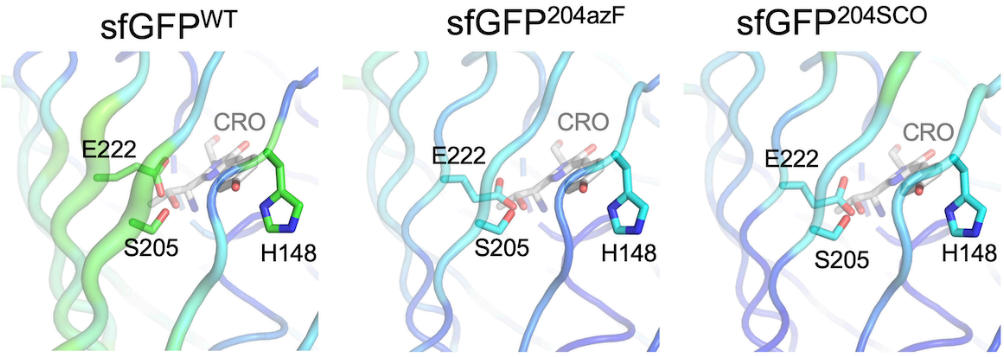

Supporting Figure S5. B-factor changes on dimerisation. B-factors are represented in the “putty” format with increased thickness corresponding to increase in B-factor and thus apparent flexibility. Shown is sfGFP^{WT} together with the monomer units (sfGFP^{204azF} and sfGFP^{204SCO}) of the sfGFP^{204x2} dimer. The regions shown comprise the dimer interface. Selected residues important for function are also shown.

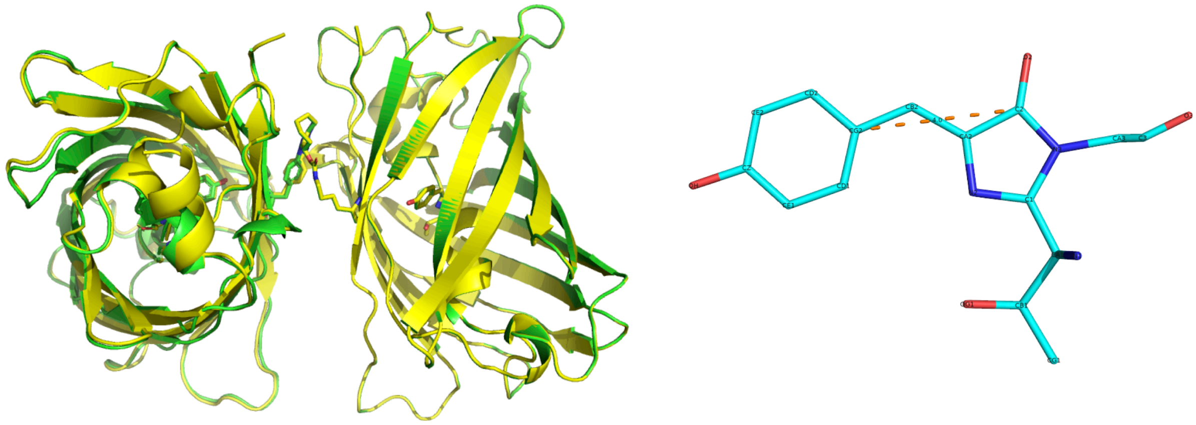

Supporting Figure S6. (a) Overlap of sfGFP^{204x2} (green) and model of the GFVen^{204} (yellow). The Cα RMSD between the structure is 0.072 Å. (b) Atomic positions in the chromophore used to define the vector the transition dipole moment for calculating κ^2

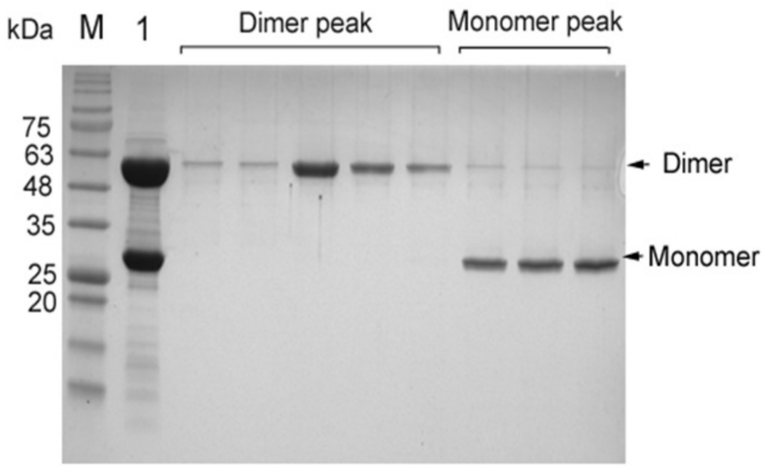

Supporting Figure S7. Elution profile of GFVen^{204} dimer and monomeric forms. SDS-PAGE analysis of elution fractions from SEC purification of GFVen^{204} dimer. Lane M corresponds to the protein marker of known molecular weights. Lane 1 corresponds to the mixture of the click dimerisation reaction before loading onto the SEC column. The dimer corresponds to the first major elution peak and monomer corresponds to the second elution peak on the chromatogram SEC profile.

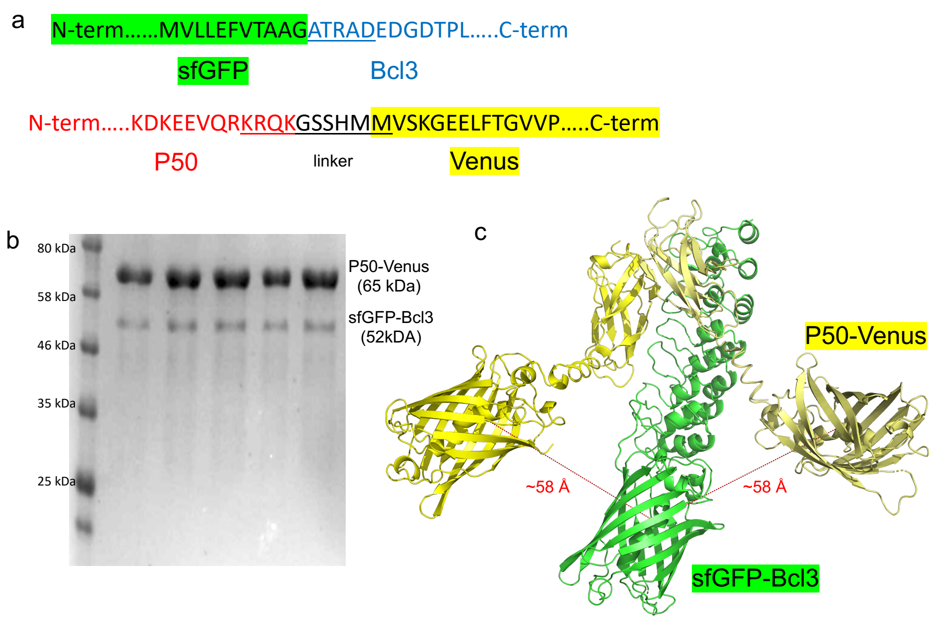

Supporting Figure S8. Complexation of sfGFP-Bcl3 with P50-Venus. (a) Fusion regions of each construct. Residues in blue, red and black represent Bcl3, P50 and artificial linker sequences, respectively. Residues underlined are not observed in crystal structures of Bcl3 or P50. The sfGFP (b) Fractions of pure complex after size exclusion chromatography. (c) Model of the P50-Venus (yellow) complexed to sfGFP-Bcl3 (green). The N-terminal DNA binding domains (present in the P50-Venus construct) have been left out for clarity. Models of each component were build using Phyre^2 (http://www.sbg.bio.ic.ac.uk/phyre2) (*10*). The models were build using overlapping sequence elements from the individual protein elements (Venus, sfGFP, P50, Bcl3). For sfGFP-Bcl3, the overlap sequence attached to sfGFP was the first ankyrin repeat sequence (ATRADEDGDTPLHIAVVQGNLPAVHRLVNLFQQGGRE)

from Bcl3. For P50-Venus, the models comprised of the P50 binding domain (residue 312 to 320) plus C-terminal residues KDKEEVQRKRQKG. The Venus model has residues EEVQRKRQKGSSHMM attached to the N-terminal of Venus. Shown in red are the estimated distances between Venus and sfGFP chromophores.

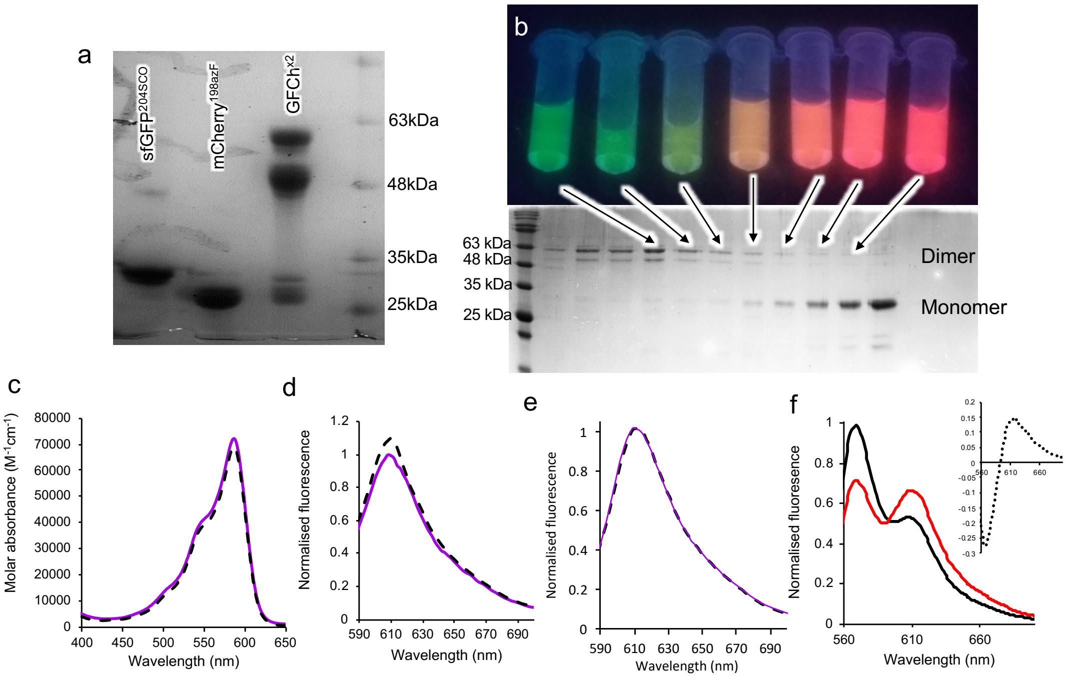

Supporting Figure S9. Dimerisation of sfGFP^{204SCO} and mCherry^{198azF}. (a) SDS PAGE analysis of the dimerisation reaction. The GFCh^{x2} lane is the endpoint of the reaction. Two bands for mCherry associated proteins have been reported previously by (*11*), which is due to hydrolysis of the chromophore on boiling. (b) Separation of the dimer from monomer. The top panel represents fluorescence output from the samples equivalent to the fractions analysed by SDS PAGE in the lower panel. On illumination with a transilluminator, the dimer fractions are green, mixed fractions are orange and mCherry based-monomers are magenta is colour. Monomer fractions equivalent to sfGFP^{204SCO} elute after mCherry (not shown). (c-f) Spectral properties of mCherry^{198azF} and various conjugates. Molar absorbance (c) and fluorescence emission on excitation at 587 nm (d) of WT mCherry (purple) and mCherry^{198azF} (black dashed lines). Fluorescence emission was normalised to WT mCherry. (e) Fluorescence emission (on excitation at 587 nm) on incubation of mCherry^{198azF} with 20 folded excess of SCO-K at the start (0 hr, black dashed lines) and after 4 hr (purple line). Fluorescence normalised to 0 hr time point. (f) Fluorescence emission (on excitation at 555 nm) on incubation of mCherry^{198azF} with DBCO-Cy3 for 0 hr (black line) and overnight (red line). Fluorescence is normalised to the 0 hr time point. Inset the subtraction spectra of the overnight sample from the 0 hr time point. The estimated *J* coupling according to FPbase between Cy3 (donor) and mCherry (acceptor) is 4.83x10^{15} M^{-1}cm^{-1}nm^{4}.

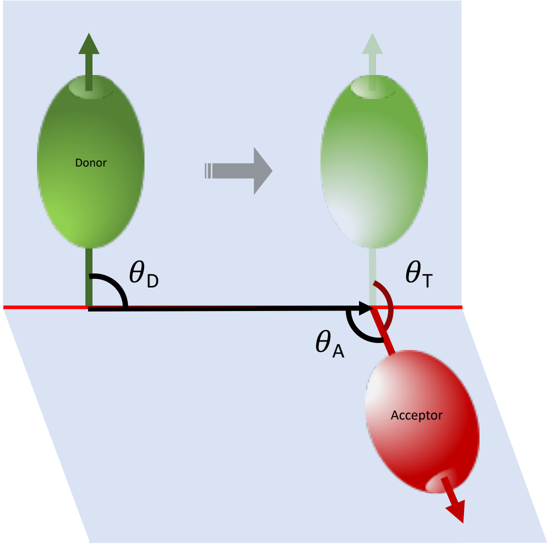

Supporting Figure S10. (a) Calculating κ^2 through chromophore dipole vector positioning using the equation $\kappa^2 = (\text{Cos}\ \theta_T - 3\text{Cos}\ \theta_D \text{Cos}\ \theta_A)^2$ [(12)]. The dipole orientations for the chromophores have been reported previously (*13, 14*) and are shown in Figures 4 and S6.

Supporting References

1. S. C. Reddington *et al.*, Genetically encoded phenyl azide photochemistry drives positive and negative functional modulation of a red fluorescent protein. *RSC Advances* **5**, 77734-77738 (2015).
2. A. M. Hartley, H. L. Worthy, S. C. Reddington, P. J. Rizkallah, D. D. Jones, Molecular basis for functional switching of GFP by two disparate non-native post-translational modifications of a phenyl azide reaction handle. *Chem Sci* **7**, 6484-6491 (2016).
3. S. C. Reddington *et al.*, Different photochemical events of a genetically encoded phenyl azide define and modulate GFP fluorescence. *Angew Chem Int Ed Engl* **52**, 5974-5977 (2013).
4. S. C. Reddington, E. M. Tippmann, D. D. Jones, Residue choice defines efficiency and influence of bioorthogonal protein modification via genetically encoded strain promoted Click chemistry. *Chemical communications* **48**, 8419-8421 (2012).
5. J. D. Pedelacq, S. Cabantous, T. Tran, T. C. Terwilliger, G. S. Waldo, Engineering and characterization of a superfolder green fluorescent protein. *Nat Biotechnol* **24**, 79-88 (2006).
6. H. L. Worthy *et al.*, Positive functional synergy of structurally integrated artificial protein dimers assembled by Click chemistry. *Communications Chemistry* **2**, 83 (2019).
7. T. Nagai *et al.*, A variant of yellow fluorescent protein with fast and efficient maturation for cell-biological applications. *Nat Biotechnol* **20**, 87-90 (2002).
8. N. C. Shaner *et al.*, Improved monomeric red, orange and yellow fluorescent proteins derived from Discosoma sp. red fluorescent protein. *Nat Biotechnol* **22**, 1567-1572 (2004).

9. E. Chovancova *et al.*, CAVER 3.0: a tool for the analysis of transport pathways in dynamic protein structures. *PLoS Comput Biol* **8**, e1002708 (2012).
10. L. A. Kelley, S. Mezulis, C. M. Yates, M. N. Wass, M. J. Sternberg, The Phyre2 web portal for protein modeling, prediction and analysis. *Nat Protoc* **10**, 845-858 (2015).
11. L. A. Gross, G. S. Baird, R. C. Hoffman, K. K. Baldridge, R. Y. Tsien, The structure of the chromophore within DsRed, a red fluorescent protein from coral. *Proc Natl Acad Sci U S A* **97**, 11990-11995 (2000).
12. B. W. van der Meer, Kappa-squared: from nuisance to new sense. *J Biotechnol* **82**, 181-196 (2002).
13. T. Ansbacher *et al.*, Calculation of transition dipole moment in fluorescent proteins--towards efficient energy transfer. *Phys Chem Chem Phys* **14**, 4109-4117 (2012).
14. A. Kyrychenko, M. V. Rodnin, C. Ghatak, A. S. Ladokhin, Joint refinement of FRET measurements using spectroscopic and computational tools. *Anal Biochem* **522**, 1-9 (2017).
